# Outcomes of Patients With Advanced Renal Cell Carcinoma With Non-Clear Cell Histology Treated With Systemic Therapy

**DOI:** 10.1016/j.clgc.2023.09.004

**Published:** 2023-09-22

**Authors:** Danielle Urman, Leah Deshler, Nicole Weise, Ahmed Shabaik, Ithaar Derweesh, Aditya Bagrodia, Brent Rose, Daniel Herchenhorn, Rana R. McKay

**Affiliations:** University of California San Diego, La Jolla, CA

**Keywords:** Immunotherapy, Nonclear cell renal cell carcinoma, Sarcomatoid, Advanced renal cell carcinomia, Metastatic renal cell carcinoma

## Abstract

This study evaluated the outcomes of patients with non-clear cell renal cell carcinoma (RCC) and RCC with sarcomatoid features receiving systemic treatment. We conducted a single-center retrospective analysis of 251 patients with advanced RCC receiving systemic therapy. Overall, patients with nonclear cell histology and RCC with sarcomatoid features had worse overall survival compared to those with clear cell histology.

**Background:**

Patients with nonclear cell renal cell carcinoma (RCC) and RCC with sarcomatoid differentiation have been under-represented in clinical trials. This study evaluates the outcomes and treatment patterns of patients with non-clear cell RCC and RCC with sarcomatoid features compared to those with clear cell RCC receiving systemic therapy.

**Methods:**

A single-center retrospective analysis of patients with advanced or metastatic RCC receiving systemic therapy was conducted. Patients were divided into groups based on histology: nonclear cell RCC, clear cell RCC, and RCC with and without sarcomatoid features. The primary endpoint was overall survival (OS) for each group calculated from the date of diagnosis of advanced or metastatic RCC to the date of last follow-up or death. Additionally, an exploratory analysis was conducted by nonclear cell type and type of first-line treatment.

**Results:**

Overall, 251 patients were included, with most treated before 2018. First-line therapies included vascular endothelial growth factor monotherapy (68.5%), immunotherapy monotherapy (7.6%), immunotherapy combination therapy (16.7%), or other treatments (7.2%). Overall survival was shorter for patients with nonclear cell RCC compared to clear cell RCC (39.2 months vs. 81.1 months, hazard ratio (HR), 1.60, 95% Confidence Interval 1.0, 2.6, *P* = .04). Additionally, OS for patients with sarcomatoid differentiation was shorter compared to patients without sarcomatoid differentiation (43.4 vs. 75.0 months, HR 1.5, 95% CI 0.8, 2.6, *P* = .20).

**Conclusion:**

We demonstrate inferior outcomes among patients with advanced or metastatic nonclear cell RCC and RCC with sarcomatoid differentiation receiving systemic treatment. Further prospective studies are warranted testing immunotherapy combinations and novel treatments in patients with nonclear cell RCC.

## Introduction

Renal cell carcinoma (RCC) is a common cancer among men and women in the United States. Renal cell carcinoma has historically been divided into clear cell and nonclear cell histologies. While historically nonclear cell RCC have been lumped into one subgroup, each entity is histologically distinct and associated with unique pathogenesis. The most common nonclear cell histologies include papillary (10%-15%), chromophobe (5%), collecting duct (1%), medullary (1%), and translocation (1%-4%). Sarcomatoid or rhabdoid differentiation can occur in any of the RCC histologic type. ^1^

Clear cell RCC represents 70% of all RCC tumors. Clear cell RCC is classically associated with mutations in the *Von Hippel Lindau* (VHL) gene located on chromosome 3p. The VHL protein is a critical player in the regulation of the hypoxia response pathway, which is vital to tumor survival. Under normoxic conditions, intact VHL protein targets hypoxia-inducible factor (HIF) for degradation. In the absence of functional VHL protein, HIF accumulates in the cell, translocates to the nucleus where it acts as a transcription factor resulting in activation of protumorogenic genes including vascular endothelial growth factor (VEGF) and other genes involved in tumor growth and survival. ^2^ Historically, most large phase 3 trials for patients with advanced or metastatic RCC have largely focused on patients with clear cell RCC and excluded patients with variant histologies.

Nonclear cell RCC represents the remaining 30% of patients with RCC tumors. The underlying molecular biology varies depending on the nonclear cell RCC subtype. Overall, in addition to the VHL pathway described above, other genes are known to contribute to the development of variant histology RCC. For example, papillary RCC is associated with aberrant MET activation. ^3^ Chromophobe RCC, the second most common nonclear cell RCC, is associated with *TP53, PTEN,* and *TERT* alterations. ^4^ While research is moving to better characterize subsets of each histologic subtype of RCC to more appropriately tailor therapy for a given patient, at the present time, the treatment paradigm for patients with variant histology RCC has largely mirrored the therapeutic strategies for patients with clear cell RCC.

Approximately, 20% of advanced RCC have sarcomatoid features, which is defined by pleomorphic spindle cells on the histopathology. The presence of sarcomatoid differentiation has historically been associated with a worse prognosis and lower response rates to standard therapy compared to clear cell RCC without sarcomatoid differentiation. ^5, 6^ Sarcomatoid RCC harbor a distinct molecular profile which likely accounts for their aggressive behavior including *BAP1* mutations, *CDKN2A* deletions, and increase expression of *MYC* transcriptional programs. ^7, 8^ More recently, these tumors have been demonstrated to be responsive to immunotherapy and immunogenomic profiling has revealed that sarcomatoid tumors exhibit an immune-inflamed phenotype characterized by immune activation, increased cytotoxic immune infiltration, upregulation of antigen presentation machinery genes, and PD-L1 expression. ^8, 9^

As the treatment options for patients with RCC have evolved over the past decade, understanding the efficacy of immunotherapy combinations and VEGF targeting agents is important for optimizing treatment selection for patients with nonclear cell RCC or RCC with sarcomatoid/rhabdoid differentiation. Given the shifting treatment landscape for patients with advanced and metastatic RCC, we aimed to characterize the outcomes of patients with variant histology RCC compared to clear cell RCC.

## Patients and Methods

### Patient Population

We conducted a retrospective single center study at the University of California San Diego. Patients with locally advanced unresectable or metastatic RCC initiating systemic therapy were consecutively and systematically captured using the electronic medical record. Patients with more than one visit at UCSD were eligible. Patients were divided into groups based on histology, nonclear cell RCC versus clear cell RCC, and presence or absence of sarcomatoid features. Pathology from patients with nonclear cell RCC underwent review by a genitourinary pathologist. This study was approved by the Institutional Review Board at the University of California San Diego.

### Clinical Data

Data collected included demographics, RCC histologic type, stage, sites of metastasis, International Metastatic RCC Database Consortium (IMDC) risk stratification, treatment regimens, and survival. Data gathered for each treatment regimen included the patient’s laboratory parameters at the start of therapy, Karnofsky performance status, type of therapy, time on therapy, and reason for treatment discontinuation.

### Outcomes

The primary outcome was overall survival (OS) for each group calculated from the time of locally advanced unresectable or metastatic cancer diagnosis to the date of last follow-up or death. The secondary endpoint was time to first treatment failure (TTF) defined as the time of systemic therapy initiation for locally advanced unresectable or metastatic cancer to treatment discontinuation for any reason, censored at the date of last treatment follow-up.

### Statistical Analysis

Kaplan-Meier (K-M) analyses were performed for OS and TTF for the overall cohort as well as between specific subgroups: clear cell RCC vs. nonclear cell RCC, presence vs. absence of sarcomatoid differentiation, among nonclear cell RCC histology groups, and nonclear cell RCC with sarcomatoid differentiation vs. clear cell RCC with sarcomatoid differentiation. Additionally, OS and TTF were evaluated in subgroups of patients receiving frontline VEGF-targeted therapy and frontline immunotherapy. Hazard ratios (HR) with the corresponding 95% confidence intervals (CI) were calculated with the Cox proportional hazards model. *P* -values were 2-sided, and *P* < .05 was considered statistically significant. Statistical analyses were generated using SAS Enterprise Guide, version 7.1.

## Results

### Baseline Characteristics

Baseline characteristics are described in Table 1. This cohort included 251 patients of whom 58 patients had variant histology RCC (n = 14 papillary, n = 8 chromophobe, n = 29 unclassified, and n = 7 translocation) and 193 had clear cell RCC. Overall, 25 patients had sarcomatoid differentiation of whom 5 were variant histology and 20 were clear cell RCC. The majority (74.9%) of patients were treated before 2018 (Table 2).

### Systemic Treatment

Systemic treatments are summarized in Table 2. First-line therapies for the overall population included VEGF monotherapy (68.5%), immunotherapy monotherapy (7.6%), immunotherapy combination treatments (16.7%), or other (7.2%). For patients with nonclear cell RCC, the most common first-line therapies included VEGF monotherapy (69.0%), immunotherapy monotherapy (6.9%), and immunotherapy combination treatments (15.5%). Overall, 25% of patients received treatment after 2018. The most common reasons for treatment discontinuation in the overall cohort included disease progression (51.2%), toxicity (28.4%), progression and toxicity (2.5%), death (6.5%), or other reasons (11.4%). The reasons for treatment discontinuation were similar across histologic subtypes (Supplemental Table 1).

### Survival Outcomes

**Figure 1:**
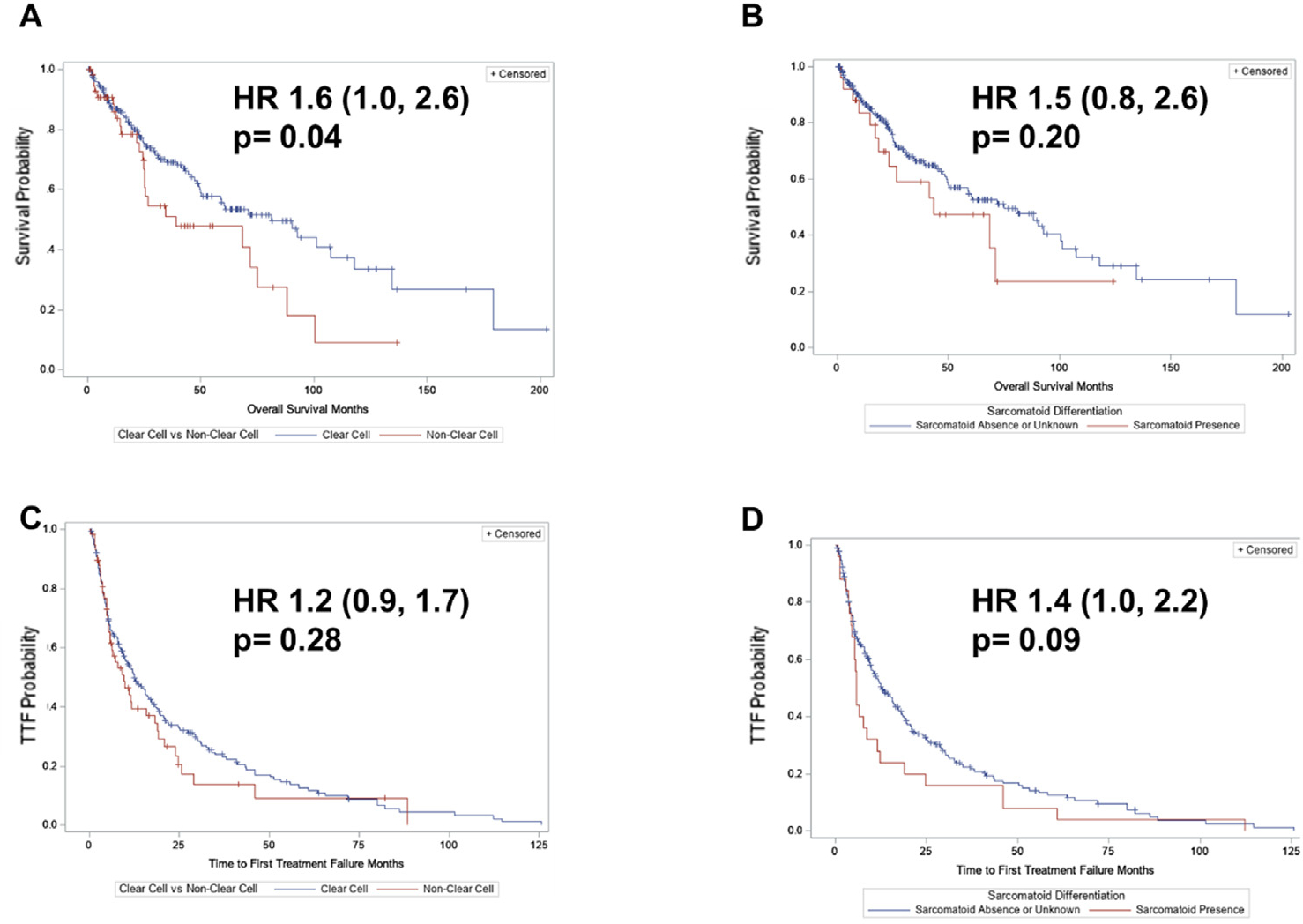
Kaplan-Meir curves comparing overall survival and time to first treatment failure in patients with various histologic subtypes. *The reference category for the Cox regressions comparing clear cell versus non-clear cell is clear cell +The reference category for the Cox regressions comparing Sarcomatoid presence versus absence is absence A. Overall survival comparing clear cell (blue) to non clear cell (red) B. Overall survival comparing absence of sarcomatoid differentiation (blue) to presence of sarcomatoid differentiation (red) C. Time to first treatment failure comparing clear cell (blue) to non-clear cell (red) D. Time to first treatment failure comparing sarcomatoid differentiation (blue) to presence of sarcomatoid differentiation (red)

Median follow-up time from advanced or metastatic disease diagnosis to death or last follow-up was 26.0 months. In the overall population, the median OS and 2-year OS from advanced or metastatic disease diagnosis was 70.9 months (95% CI 49.6, 92.8) and 75.8% (95% CI 69.4%, 81.0%), respectively. Overall survival from advanced or metastatic disease was shorter among patients with nonclear cell RCC compared to clear cell RCC (median 39.2 months vs. 81.1 months; HR 1.65 95% CI 1.0, 2.6, *P* = .04) (Table 3; Figure 1).

For the total cohort, the median TTF for first-line therapy was 11.9 months (95% CI 9.4, 15.6). Time to first-line treatment failure was numerically but not statistically shorter between nonclear cell RCC patients compared to patients with clear cell RCC (median 9.5 months vs. 12.4 months, HR 1.20, 95% CI 0.9, 1.7, *P* = .280) (Table 3; Figure 1).

When evaluating the impact of sarcomatoid differentiation on outcomes, we observed numerically shorter OS from advanced or metastatic disease (median 43.4 months vs. 75.0 months, HR 1.5, 95% CI 0.8, 2.6, *P* = .20) and first-line TTF (median 5.8 months vs. 12.8 months, HR 1.45, 95% CI 1.0, 2.21, *P* = .09), though the results were not statistically significant (Table 3; Figure 1).

We conducted an exploratory analysis evaluating OS from advanced or metastatic disease diagnosis and TTF by nonclear cell type and first-line treatment type. The median OS for patients with papillary, chromophobe, translocation, and unclassified RCC, respectively, was 75.0 months (95% CI 12.3, 88.3), 25.9 months (95% CI 24.4, 72.0), 68.3 months (95% CI 11.3, 100.5), and 34.3 (95% CI 14.2, Not Reached) months (Table 3). The median first-line TTF for patients with papillary, chromophobe, translocation, and unclassified RCC, respectively, was 11.0 months (95% CI 5.1, 19.2), 13.8 (1.6, 20.8), 15.8 months (95% CI 2.2, 45.9), and 6.4 months (95% CI 4.8, 23.9) (Table 3). The median OS and TTF were shorter among nonclear cell RCC patients compared to clear cell RCC patients receiving either VEGF-targeted therapy or front-line immunotherapy (Table 4).

## Discussion

While the treatment options for clear cell RCC have largely expanded over the past decade, data regarding the efficacy of such treatments in patients with nonclear cell RCC are limited. ^10, 11^ Additional data on the outcomes of patients with nonclear cell RCC receiving systemic therapy are needed, specifically with respect to immunotherapy-based treatment. This real-world study characterizes the treatment patterns for patients with nonclear cell RCC during the shift in the RCC treatment paradigm to frontline immunotherapy combination for patients with advanced disease. We investigate outcomes in patients with nonclear cell histologic types receiving immunotherapy and VEGF-targeted therapy.

Consistent with other studies, we demonstrate inferior OS in patients with nonclear cell RCC compared to clear cell RCC. ^5, 6^ Additionally, while our sample size is limited, we show survival outcomes are inferior for patients with RCC with sarcomatoid features compared to those without sarcomatoid features, also consistent with prior trials. ^5, 6, 12^ The overall survival for patient’s with clear cell RCC receiving any type of frontline therapy was 81.1 months. This is much longer than other similar phase 3 trials.^10,11, 13, 14^ Though 2-year overall survival for all patients was 75.8% (95% CI 69.4%, 81.0%) consistent with other phase 3 trials with VEGF predominant treatment. ^15, 16^ This could be because, in our retrospective study, we included all patients initiating systemic treatment for a metastatic or advanced disease that had more than 1 consultation at UCSD, however not all patients continued therapy at our institution and therefore there could be ascertainment bias. Our cohort included 24% of patients treated with immunotherapy-based treatment. While historical data on patients receiving VEGF monotherapy has shown consistent results, recent data does show improvement in outcomes with immunotherapy combination treatments. ^14^

With the shift in the treatment landscape for frontline clear cell RCC, several single arm phase 2 trials have investigated the activity of immunotherapy combination regimens in patients with nonclear cell RCC (Table 5). The COSMIC-021 study investigated the combination of cabozantinib plus atezolizumab and demonstrated and objective response rate of 31%. ^17^ Lee et al. ^18^ investigated the activity of nivolumab plus cabozantinib in patients with nonclear cell RCC. This study had two cohorts including cohort 1 for patients with papillary, unclassified, or translocation RCC and cohort 2 for chromophobe RCC. Patients enrolled on cohort 1 demonstrated an objective response rate of 47.5%, while no responses were observed in cohort 2 and the arm was closed for futility at the interim analysis. Similar responses were observed with the combination of lenvatinib plus pembrolizumab in patients with treatment naïve nonclear cell RCC in the Keynote B61 trial (objective response rate 47.6%). ^19^ Another phase 2 study observed responses to atezolizumab combined with bevacizumab in patients with nonclear cell RCC or with more than 20% sarcomatoid differentiation. This study showed an objective response rate of 33%. ^20^ Several small phase 2 studies have also investigated pure immunotherapy regimens, either as monotherapy or combined with checkpoint inhibition, in patients with nonclear cell RCC. In aggregate, these studies demonstrated a modest objective response rates ranging from 14.3% to 26.7%. ^21, 22^

As our understanding of the pathogenesis and molecular under-pinning for nonclear cell RCC expands, better tailoring therapy to target key pathogenic drivers of disease for patients with nonclear cell RCC is warranted. Papillary RCC represents a model RCC subtype in which precision medicine strategies have advanced outcomes. Understanding that papillary RCC is largely driven by alterations in the MET pathway, the PAPMET trial investigated the efficacy of cabozantinib, savolitinib, crizotinib, versus sunitinib in patients with papillary RCC. ^23^ This was a positive trial demonstrating improvements in radiographic progression-free survival (9.0 vs. 5.6 months) and overall response rates (23% vs. 4%) with cabozantinib compared to sunitinib in patients with papillary RCC. Additionally, the SAVIOR trial, comparing savolitinib to sunitinib, was specifically conducted in patients with MET-driven tumors. ^24^ While the study was closed early, numerically improved progression-free survival and objective responses were seen with savolitinib (progression-free survival 7.0 months vs. 5.6 months; objective response rate 27% vs. 7%). Currently, the PAPMET2 trial is investigating the role of atezolizumab plus cabozantinib compared to cabozantinib alone in patients with papillary RCC (NCT05411081). ^25^

Chromophobe RCC has proven to be a tumor more resistant to standard RCC treatments. In cohort 2 of the phase 2 study evaluating nivolumab plus cabozantnib enrolling patients with chromophobe RCC, the response rate was 0% and the arm was closed for futility. The combination of lenvatinib and everolimus has demonstrated clinical efficacy for patients with chromophobe RCC. In a small phase 2 study of lenvatinib plus everolimus in patients with non-clear cell RCC, partial responses were observed in 4/9 patients (44%) of patients with chromophobe RCC. ^26^ Overall outcomes remain poor for patients with chromophobte RCC and alternative treatment strategies are warranted for this tumor type.

Historically, during the VEGF targeted therapy era, patients with sarcomatoid features had poor responses and inferior outcomes to systemic therapy. As immunotherapy combinations have populated the front-line space for patients RCC, we have observed improved response rates and durable clinical benefit with immunotherapy-based strategies. Long-term data from the Checkmate-214 trial of the combination of nivolumab and ipilimumab compared to sunitinib for patients with intermediate/poor risk disease with sarcomatoid RCC demonstrate increased objective response rate (60.8% vs. 23.1%), improved progression-free survival (26.5 months vs. 5.5 months) and improved OS (48.6 vs. 14.2 months). ^9^ Furthermore, at a minimum of 5 years of follow-up data, 12% of patients remained on treatment without disease progression with nivolumab and ipilimumab. Based on the results of this study, the combination of nivolumab and ipilimumab has become a preferred regimen for patients with RCC with sarcomatoid differentiation. ^9,27–29^

The major limitation of this study is that many of the patients in the database received treatment prior to 2018 when the standard of care shifted from VEGF monotherapy to combination therapy. The majority of the patients received VEGF therapy alone. This reflects historic treatment patterns versus outcomes with respect to what is the current standard of care. Additionally, the IO regimens in this paper largely do not reflect modern practice and results cannot be extrapolated to inform clinical decision making. Given the differences in follow-up durations for patients receiving treatment in the targeted therapy era (2018 and prior) and those receiving treatment in the combination era (post-2018), we excluded this analysis from the manuscript. Furthermore, due to its retrospective design, this study was limited by the small sample size of each of the distinct variant RCC subtypes. There were also limitations regarding missing clinical data.

## Conclusion

In this institutional dataset, we demonstrate inferior outcomes among patients with nonclear cell RCC receiving tyrosine kinase inhibitors and immunotherapies as first line systemic therapy. Further prospective studies testing combination and novel treatment strategies are warranted for patients with nonclear cell RCC. Additionally, improved understanding of the disease pathogenesis will be critical to improving treatment selection for patients with variant histology RCC.

## Supplementary Material

1

## Figures and Tables

**Table 1:** Baseline Characteristics of the Overall Cohort at Time of Systemic Therapy Initiation

	Clear Cell n = 193 (%)	Nonclear Cell n = 58 (%)	*P*-Values	Sarcomatoid Differentiation Present n = 25 (%)	Sarcomatoid Differentiation Absent Or Unknown n = 226 (%)	P-Values
Age at diagnosis, median years (IQR)	61 (15)	60 (24)	.038	65 (14)	60 (17)	.293
Gender			.343			.878
Female	52 (26.9)	20 (34.5)		8 (32.0)	64 (28.3)	
Race			.005			.744
White	104 (53.9)	35 (60.3)		15 (60.0)	124 (54.9)	
Black	7 (3.6)	3 (5.2)		1 (4.0)	9 (4.0)	
Asian or Pacific Islander	12 (6.2)	11 (19.0)		1 (4.0)	22 (9.7)	
Other or Mixed Race	66 (34.2)	9 (15.5)		7 (28.0)	68 (30.1)	
Unknown	4 (2.1)	0 (0.0)		1 (4.0)	3 (1.3)	
Ethnicity			.001			.567
Hispanic	60 (31.1)	5 (8.6)		7 (28.0)	58 (25.7)	
Nonclear cell histology			<. 001			.890
No	193 (100.0)	0 (0.0)		20 (80.0)	173 (76.6)	
Yes	0 (0.00)	58 (100.0)		5 (20.0)	53 (23.5)	
Papillary	0 (0.00)	14 (24.1)		0 (0.0)	14 (6.2)	
Chromophobe	0 (0.00)	8 (13.8)		1 (4.0)	7 (3.1)	
Collecting duct	0 (0.00)	0 (0.0)		0 (0.0)	0 (0.0)	
XpTranslocation	0 (0.00)	7 (12.1)		1 (4.0)	6 (2.7)	
Unclassified	0 (0.00)	29 (50.0)		3 (12.0)	26 (11.5)	
Presence of sarcomatoid dedifferentiation			.508			<. 001
Yes	20 (10.4)	5 (8.6)		25 (100.0)	0 (0.0)	
Unknown	93 (48.2)	33 (56.9)		0 (0.0)	126 (55.8)	
Prior nephrectomy			.174			.462
Yes	143 (74.1)	37 (63.8)		20 (80.0)	160 (70.8)	
T stage at diagnosis			.321			.002
T0	1 (0.5)	0 (0.0)		0 (0.0)	1 (0.4)	
T1	20 (10.4)	3 (5.2)		1 (4.0)	22 (9.7)	
T2	26 (13.5)	7 (12.1)		0 (0.0)	33 (14.6)	
T3	78 (40.4)	18 (31.0)		18 (72.0)	78 (34.5)	
T4	10 (5.2)	4 (6.9)		3 (12.0)	11 (4.9)	
Unknown	58 (30.1)	26 (44.8)		3 (12.0)	81 (35.8)	
N stage at diagnosis			.303			.036
N0	46 (23.8)	13 (22.4)		7 (28.0)	52 (23.0)	
N1	27 (14.0)	13 (22.4)		8 (32.0)	32 (14.2)	
Unknown	120 (62.2)	32 (55.2)		10 (40.0)	142 (62.8)	
M stage at diagnosis			.270			.335
M1	52 (26.9)	17 (29.3)		10 (40.0)	59 (26.1)	
≥1 site of metastasis			.318			.534
No	76 (39.4)	29 (50.0)		8 (32.0)	97 (42.9)	
Yes	116 (60.1)	29 (50.0)		17 (68.0)	128 (56.6)	
Unknown	1 (0.5)	0 (0.0)		0 (0.0)	1 (0.4)	
IMDC risk groups			.504			.253
Favorable	26 (13.5)	6 (10.3)		1 (4.0)	31 (13.7)	
Intermediate	86 (44.6)	24 (41.4)		12 (48.0)	98 (43.4)	
Poor	48 (24.9)	13 (22.4)		9 (36.0)	52 (23.0)	
Unknown	33 (17.1)	15 (25.9)		3 (12.0)	45 (19.9)	
Site of metastasis at diagnosis of metastatic disease			.595			.409
Bone	6 (3.1)	3 (5.2)		0 (0.0)	9 (4.0)	
Brain	1 (0.5)	1 (1.7)		0 (0.0)	2 (0.9)	
Liver	2 (1.0)	1 (1.7)		1 (4.0)	2 (0.9)	
Lung	47 (24.4)	12 (20.7)		9 (36.0)	50 (22.1)	
Multiple	93 (48.2)	22 (37.9)		9 (36.0)	106 (46.9)	
Other	22 (11.4)	9 (15.5)		2 (8.0)	29 (12.8)	
None	22 (11.4)	10 (17.2)		4 (17.0)	28 (12.4)	
						

IQR = interquartile range; IMDC = International Metastatic RCC Database Consortium.

**Table 2:** Summary of Year of Initiation and Type of First-line Systemic Therapy

	Clear Cell N (%) n = 193	Nonclear Cell N (%) n = 58	Sarcomatoid Differentiation Present N (%) n = 25	Sarcomatoid Differentiation Absent Or Unknown N (%) n = 226
Year of Systemic Therapy Initiation				
2005 and prior	3 (1.6)	0 (0.0)	0 (0.0)	3 (1.3)
2006–2018	142 (73.6)	43 (74.1)	20 (80.0)	165 (73.0)
After 2018	48 (24.9)	15 (25.9)	5 (20.0)	58 (25.7)
Type of First Line Systemic Therapy				
TKI	132 (68.4)	40 (69.0)	19 (76.0)	153 (67.7)
TKI + TKI	0 (0.0)	1 (1.7)	0 (0.0)	1 (0.4)
IO	15 (7.8)	4 (6.9)	1 (4.0)	18 (8.0)
IO+1 O	33 (17.1)	9 (15.5)	3 (12.0)	39 (17.3)
IO+TKI	5 (2.6)	3 (5.2)	2 (8.0)	6 (2.7)
Other	8 (4.2)	1 (1.7)	0 (0.0)	9 (4.0)

IO = immunooncology; TKI = tyrosine kinase inhibitor.

**Table 3:** Time to First-line Treatment Failure and Overall Survival of Patients With Advanced or Metastatic RCC

	Time to First-line Treatment Failure				Overall Survival			

	Number of Patients n = 251	Median (95% CI), months	HR (95% CI)	*P*-Value	Number of Patients n = 251	Median (95% CI), months	HR (95% CI)	*P*-value
Overall	201	11.9 (9.4, 15.6)	-	-	99	70.9 (49.6, 92.8)	-	-
Histology								
Clear cell	159	12.4 (9.4, 16.6)	1.2 (0.9, 1.7)	.28^a^	74	81.1 (50.0, 107.2)	1.6 (1.9, 2.6)	**.04** ^a^
Nonclear cell	42	9.5 (5.7, 18.2)			25	39.2 (24.8, 75.0)		
Nonclear cell Histology								
Papillary	10	11.0 (5.1, 19.2)	-	-	6	75.0 (12.3, 88.3)	-	-
Chromophobe	7	13.8 (1.6, 20.8)	-	-	5	25.9 (24.4, 72.0)	-	-
XpTranslocation	6	15.8 (2.2, 45.9)	-	-	4	68.3 (11.3, 100.5)	-	-
Unclassified	19	6.4 (4.8, 23.9)	-	-	10	34.3 (14.2, INF)	-	-
Sarcomatoid Differentiation								
Overall, Absent	25	5.8 (4.5, 11.4)	1.4 (1.0, 2.2)	.09^b^	13	43.4 (18.6, INF)	1.5 (0.8, 2.6)	.20^b^
Overall, Present	176	12.8 (9.8, 17.2)			86	75.0 (50.0, 100.5)		

CI = confidence i nterval; HR = hazard ratio.

Bolded values are statistically significant.

a 7 observations with missing metastasis diagnosis date were removed from overall survival analysis.

b The reference category for the Cox regressions comparing clear cell vs. nonclear cell is clear cell, The reference category for the Cox regressions comparing sarcomatoid presence vs. absence i s absence.

**Table 4:** Treatment Outcomes of Patients With Advanced Renal Cell Carcinoma Receiving First-line Tyrosine Kinase Inhibitors or Immunotherapy

	Overall Survival (OS)						Time to First-line Treatment Failure (TTF)					

	Tyrosine Kinase Inhibitor (TKI)		Immunotherapy (IO)				Tyrosine Kinase Inhibitor (TKI)		Immunotherapy (IO)			

	Number of Patients n = 173	Median (95% Cl), months	Number of Patients n = 69	Median (95% Cl), months	HR (95% Cl)	P-Value	Number of Patients n = 173	Median (95% Cl), months	Number of Patients n = 69	Median (95% Cl), months	HR (95% Cl)	P-Value
Overall	73	70.9 (50.0, 92.8)	23	29.3 (22.5, INF)	-	-	144	12.5(9.8,17.2)	50	7.2 (4.8, 15.0)	-	-
Clear cell	54	81.1 (50.0, 117.8)	17	Not Reached	1.6(1.0,2.5)	.08^a^	114	13.5(10.3,18.0)	38	9.3 (3.9, 16.6)	1.4 (1.0,1.9)	.05^a^
Nonclear cell	19	68.3 (24.8, 88.3)	6	25.0 (11.5,26.7)			30	11.0(5.3,19.2)	12	6.5 (4.9, 19.0)		

Cl = confidence Interval; 10 = Immunooncology; OS = overall survival; TTF = tlme-to-treatmentfailure; TKI = tyrosine kinase Inhibitor.

a The reference category for the Cox regressions comparing TKI subset vs. 10 subset Is TKI subset.

**Table 5:** Outcomes of Clinical Trials Investigating Patients With Nonclear cell RCC

Study	Phase	Agent	N	Histology	ORR (%)	PFS (months)
VEGF Monotherapy						
PapMet	2	Cabozantinib vs. Sunitinib	147	Papillary	23% vs. 4%	9.0 vs. 5.6
Savior	3	Savolitinib vs. Sunitinib	60	Met-driven RCC	27% vs. 7%	7.0 vs. 5.6
Immunotherapy						
Keynote-427	2	Pembrolizumab	165	Papillary, chromophobe, unclassified	26.7%	4.2
HCRN GU16–260	2	Nivolumab	35	Papillary, chromophobe, unclassified	14.3%	4.0
Checkmate-920	2	Nivolumab + Ipilimumab	52	Non-clear cell RCC	19.6%	3.7
Immunotherapy + VEGF						
Cosmic-021	1b	Cabozantinib + Atezolizumab	32	Nonclear cell RCC	31%	9.5
Lee, JCO, 2022	2	Nivolumab + Cabozantinib	47	Cohort 1) Papillary, unclassified, or translocation; 2) Chromophobe	1) 47.5%; 2) 0%	1) 12.5
Albiges, ESMO, 2022	2	Pembrolizumab + Lenvatinib	82	Non-clear cell RCC	47.6%	72.3%^a^
McGregor, JCO, 2020	2	Atezolizumab + Bevacizumab	60	Non-clear cell RCC; >20% Sarcomatoid	33%	8.3

a 6-month progression-free survival rate.

## Clinical Practice Points

- It is well known that there are inferior outcomes among patients with advanced or metastatic nonclear cell RCC and RCC with sarcomatoid differentiation receiving systemic treatment.
- Treatment options for clear cell RCC have greatly expanded in recent years.
- There is a paucity of survival data for patients with nonclear cell RCC receiving new treatment options, especially immunotherapies.
- We demonstrate inferior outcomes among patients with nonclear cell RCC receiving tyrosine kinase inhibitors and immunotherapies as first-line systemic therapy. This warrants further studies for testing combination and novel treatment strategies.
