# Supplementary material for: Outcomes of Patients With Advanced Renal Cell Carcinoma With Non-Clear Cell Histology Treated With Systemic Therapy
Source: Clin Genitourin Cancer. Author manuscript; Available in PMC 2026 Aug 9. (PMC13453288; doi:10.1016/j.clgc.2023.09.004)
Supplement: Supplementary file 1 [file NIHMS1970204-supplement-1.pdf]

Supplementary materials

Supplement Table 1

Supplement Table 1 Reason for First-line Treatment Discontinuation by Treatment Regimen and by Histology

Treatment Regimen	Progression N (%)	Toxicity N (%)	Progression and Toxicity N (%)	Death N (%)	Other N (%)
Therapy Type					
IO	6 (40.0)	5 (33.3)	0 (0.0)	1 (6.7)	3 (20.0)
IO+IO	15 (51.7)	8 (27.6)	2 (6.9)	3 (10.3)	1 (3.5)
IO+TKI	4 (66.7)	2 (33.3)	0 (0.0)	0 (0.0)	0 (0.0)
Other	4 (57.1)	3 (42.9)	0 (0.0)	0 (0.0)	0 (0.0)
TKI	74 (51.8)	39 (27.3)	2 (1.4)	9 (6.3)	19 (13.3)
TKI+TKI	0 (0.0)	0 (0.0)	1 (100.0)	0 (0.0)	0 (0.0)
Histology Type					
Non-clear cell RCC	22 (52.4)	10 (23.8)	3 (7.1)	3 (7.1)	4 (9.5)
Clear cell RCC	81 (50.9)	47 (29.6)	2 (1.3)	10 (6.3)	19 (12.0)
Sarcomatoid absent	92 (52.3)	48 (27.3)	5 (2.8)	12 (6.8)	19 (10.8)
Sarcomatoid present	11 (44.0)	9 (36.0)	0 (0.0)	1 (4.0)	4 (16.0)

IO = Immunooncology; RCC = Renal cell carcinoma; TKI = Tyrosine kinase inhibitor.
